# Home blood pressure measurement for hypertension management in the real world: Do not just measure, but share with your physician

**DOI:** 10.3389/fcvm.2023.1103216

**Published:** 2023-01-18

**Authors:** Moo-Yong Rhee, Masanori Munakata, Deuk-Young Nah, Je Sang Kim, Hae-Young Kim

**Affiliations:** ^1^Cardiovascular Center, Dongguk University Ilsan Hospital, Goyang-si, Gyeonggi-do, Republic of Korea; ^2^College of Medicine, Dongguk University, Gyeongju-si, Gyeongsangbuk-do, Republic of Korea; ^3^Division of Hypertension and Research Center for Lifestyle-Related Disease, Tohoku Rosai Hospital, Sendai, Japan; ^4^Cardiovascular Center, Dongguk University Gyeongju Hospital, Gyeongju-si, Gyeongsangbuk-do, Republic of Korea; ^5^Department of Health Policy and Management, College of Health Science and Department of Health Care Sciences, Graduate School and BK21 FOUR R&E Center for Learning Health Systems, Korea University, Seoul, Republic of Korea

**Keywords:** blood pressure, home, ambulatory, hypertension, treatment

## Abstract

**Introduction:**

Studies of the effectiveness of home blood pressure (BP) measurement on the treatment of hypertension in the real world are sparse, and the results are controversial. There is an efficacy-effectiveness gap in the treatment of hypertension using home BP measurements. We aimed to investigate the effect of reporting home BP to physicians on ambulatory BP control as a factor contributing to the efficacy-effectiveness gap in treating patients with hypertension.

**Methods:**

We recruited patients ≥20 years of age taking antihypertensive drugs. Office and 24-h ambulatory BP were measured. A questionnaire to the measurement of home BP was conducted. Participants were divided into an HBPM(−) group, home BP was not measured (*n* = 467); HBPM(+)-R(−) group, home BP was measured but not reported (*n* = 81); and HBPM(+)-R(+) group, home BP was measured and reported (*n* = 125).

**Results:**

The HBPM(+)-R(+) group had significantly lower office systolic BP (SBP, *p* = 0.035), 24-h SBP (*p* = 0.009), and daytime SBP (*p* = 0.016) than the HBPM(−) group, and lower nighttime SBP (*p* = 0.005) and diastolic BP (DBP, *p* = 0.008) than the HBPM(+)-R(−) group. In the multivariate analysis, the differences in 24-h SBP, daytime SBP, and nighttime DBP remained significant. There was a significant difference between groups in the target achievement rate of 24-h SBP (*p* = 0.046), nighttime SBP (*p* = 0.021), and nighttime DBP (*p* = 0.023). The nighttime SBP and DBP target achievement rates in the HBPM(+)-R(+) group were higher than those in the HBPM(+)-R(−) group (*p* = 0.006 and 0.010, respectively). Among patients measuring home BP, the adjusted odds ratio for 24-h and nighttime BP target achievement in the HBPM(+)-R(+) group were 2.233 and 3.658, respectively.

**Conclusion:**

Home BP measurements should be reported to the treating physician to effectively manage hypertension.

**Clinical trial registration:**

https://clinicaltrials.gov, identifier NCT03868384.

## Introduction

Hypertension is a leading and modifiable risk factor for cardiovascular mortality and morbidity globally (1). Its global prevalence has not changed, although it had declined in some countries, because it is unchanged or increased in middle- and low-income countries (2). Despite numerous studies and efforts to treat hypertension and reduce complications, the burden of hypertension has not declined. In Korea, the prevalence modestly decreased in 2018, but the absolute number of people with hypertension has steadily increased with the rapid aging of the population (3). Therefore, prevention, diagnosis, and control of hypertension are essential to reduce the cardiovascular burden of hypertension. However, hypertension management (awareness, treatment, and control rates) has plateaued since 2007 in Korea although it has improved extensively over the past two decades (3).

Many studies have shown that home blood pressure (BP) has more substantial predictive power for cardiovascular morbidity and mortality than office BP (4, 5) and is effective in detecting white-coat and masked hypertension at a low cost (6, 7). The efficacy of home BP measurement in reducing BP has also been demonstrated in many prospective randomized controlled studies (8–10) and meta-analyses (11–13). Therefore, many guidelines recommend home BP measurement as an effective modality for the diagnosis and treatment of hypertension (6, 7, 14).

However, studies of the effectiveness of home BP measurement on the treatment of hypertension in the real world are sparse, and the results were controversial (15, 16). There is an efficacy-effectiveness gap in the treatment of hypertension using home BP measurements (17). The representative factors contributing to this gap include provider and patient factors (18). As per the provider factors, the attitude of physicians served as a well-known obstacle to implementing home BP measurement in diagnosing and treating hypertension in the real world (15, 16, 19–21). For the patient factors, some patients do not measure home BP according to guidelines recommendations, report their measured home BP to their physicians, or receive education on how to measure home BP (19, 22). Unlike in controlled studies, if measured home BP is not reported to physicians, it cannot be reflected in the treatment even though it was appropriately measured. Although many guidelines emphasize and recommend its importance in the diagnosis and treatment of hypertension (6, 7, 14, 23), physicians and patients are under-educated due to a lack of understanding of real-world situations. Therefore, it is necessary to study and understand home BP measurements and their appropriate use in the real world.

This study aimed to investigate the impact of measuring and reporting home BP to physicians on the control of 24-h ambulatory BP as a factor contributing to the efficacy-effectiveness gap in hypertensive patients treated with antihypertensive drugs.

## Materials and methods

### Study subjects

This was a cross-sectional community-based study. We recruited 701 participants between August 2018 and April 2020. The sample size of the study was calculated based on a previous controlled study, where the daytime BP control rate was 62% for care with home BP and 50% for that with office BP (10). We assumed the rate of home BP measurement in the real world to be 30%. A sample size of 446 for the group of patients who did not measure home BP and 192 for the group of patients who measure home BP by an allocation ratio of 7:3 was calculated as sufficient to have 80% power with a 0.05 two-sided significance level. Unlike the previous controlled trial (10), this was an observational study in the real world. Therefore, we assumed that more subjects must be enrolled and set the anticipated target number for this study as 1,000. However, the recruitment of participants was terminated early owing to the COVID-19 pandemic.

We recruited research participants *via* advertisements in Goyang-si and Paju-si, Gyeonggi Province, South Korea. Postcards stating the research title, purpose, and eligibility criteria were sent to each household. Posters were attached to hospitals, public health centers, and social facilities to recruit volunteers.

Patients taking antihypertensive medication for more than one year and only those ≥20 years of age were recruited for this study. The exclusion criteria were as follows: secondary hypertension; hypertensive emergency disease (hypertensive emergency or urgency); heart failure (III-IV according to NYHA functional classification); diagnosis of cardiovascular disease within six months; clinically significant arrhythmias (ventricular tachycardia, atrial fibrillation, atrial flutter, and arrhythmias determined by the investigator to be ineligible for participation in a clinical study); participation in other clinical research and taking clinical trial drugs within the last month; a history of drug or alcohol dependence within 6 months; and diseases or conditions that could, in the opinion of the investigator, interfere with the completion of the study.

### Study protocol

Volunteers were recruited after telephone or face-to-face interviews to explain the study’s objectives, process, and eligibility for the study. On the first day of the visit, the office BP of each arm was measured simultaneously at the clinical trial center, and a questionnaire for the measurement of home BP was conducted. After the survey, participants wore an ambulatory BP monitor. The next day, the patient visited the clinical trial center, the 24-h ambulatory BP monitor was removed, and the office BP of each arm was measured. They visited the clinical trial center a few days later, and the office BP of each arm was measured.

Fasting blood glucose, HbA1c, total cholesterol, triglyceride, high-density lipoprotein cholesterol, low-density lipoprotein cholesterol, blood urea nitrogen, creatinine, serum sodium and potassium levels, and urine microalbumin level were measured after at least 8 h of overnight fasting. A 12-lead resting electrocardiography was recorded.

Written informed consent was obtained from all the participants prior to their enrollment into the study. The study protocols and informed consent forms were reviewed and approved by the Institutional Review Board of Dongguk University Ilsan Hospital (DUIH 2018-02-013-002). This study was registered on the ClinicalTrial.gov website (registration no: NCT03868384).

### Measurement of office and ambulatory BP

Attended office BP was measured by a trained nurses in a quiet room at each visit. We used validated oscillometric device (WatchBP Office; Microlife, Taiwan) which can measure BP in both arms simultaneously. After 5 min of seated rest with appropriate size cuff, BPs were measured 3 time at 1-min interval. Participants were asked to avoid smoking, caffeine-containing beverages, and exercise within 30 min preceding the measurements. The nine office BP readings (3 readings at every 3 visits) of each arm were averaged, and the BP of the arm with the higher average BP was used as the office BP of the index arm.

Ambulatory BP monitoring over 24–25 h was performed on the non-dominant arm using an automated, non-invasive oscillometric device (Mobil-O-Graph, I.E.M GmbH, Stolberg, Germany), with a measurement interval of 30 min. The participants were instructed to continue normal daily activities. Valid readings for >70% of the total measurement attempts, at least 14 measurements during the fixed daytime (09:00–21:00), and at least seven measurements during the fixed nighttime (00:00–06:00) were defined as valid measurements.

### Definition of target BP achievement

The 2018 European Society of Cardiology/European Society of Hypertension (ESC/ESH) and 2018 Korean Society of Hypertension guidelines set the target BP threshold differently according to age and comorbidities (7, 14). However, the different target BP thresholds are complicated in clinical practice, and both guidelines recommend SBP of <130 mmHg or up to 130 mmHg. The 2018 ESC/ESH guidelines set a DBP of 70–79 mmHg. Therefore, we defined target BP achievement rate as a percentage of patients who achieved a target BP based on the definition suggested by the 2017 American College of Cardiology/American Heart Association (ACC/AHA) hypertension guidelines (6). The target office BP was SBP < 130 mmHg and DBP < 80 mmHg. The target ambulatory BP was 24-h SBP < 125 mmHg and DBP < 75 mmHg, daytime SBP < 130 mmHg and DBP < 80 mmHg, and nighttime SBP < 110 mmHg and DBP < 65 mmHg. The ambulatory BP targets were determined as the BP levels corresponding to the target office BP of the 2017 ACC/AHA guidelines (6).

### Survey for the status of home BP measurements

The questionnaire inquired about the duration of taking antihypertensive medications, name of the antihypertensive drugs, types of clinics and physicians prescribing hypertension treatment (primary, secondary, and tertiary hospitals, specialty of physicians), smoking status, alcoholic beverage drinking status, whether they are doing diet control and exercise, type of exercise, and duration of exercise per week. Total daily alcohol consumption (g/day) was calculated as follows: drinking days per week × number of glasses in one sitting × 7 g (amount of alcohol contained in one standard glass). Concerning home BP measurement, single-choice, multiple-choice, or open-ended questions were asked (Supplementary material).

### Statistical analysis

Patients who measured their home BP at least one day or more per month were considered to measure home BP (24). Those who measured their home BP for more than 6 months from the survey date were considered to have effective home BP measurement for the treatment of hypertension. We defined measurements more than 6 months before the survey and more than one day a month as appropriate home BP measurements. Based on the definition of proper home BP measurement, the patients were divided into two groups. Patients who did not measure home BP, measured it within 6 months from the survey date, or less than one day per month were classified as HBPM(−) group, and patients who measured home BP properly based on our definition were classified as HBPM(+). The HBPM(+) group was divided into the HBPM(+)-R(−) group (patients who had measured home BP for more than 6 months from the survey date and one day or more per month but did not report their home BP to their physicians) and HBPM(+)-R(+) group (patients who had measured home BP for more than 6 months from the survey date and one day or more per month and reported their home BP to their physicians). Statistical analyses were conducted to make appropriate comparison between the HBPM(−) and HBPM(+) groups as well as between the HBPM(−), HBPM(+)-R(−), and HBPM(+)-R(+) groups.

All data are presented as numbers (percentages) or means ± standard deviations. Comparisons of baseline clinical characteristics between groups were performed using the unpaired *t*-test comparison of the two groups (continuous variables), Chi-square test (categorical variables), or the analysis of variance for comparison of three groups (continuous variables).

Comparisons of BP were conducted by analysis of variance (ANOVA) with Tukey’s or Games–Howell *post-hoc* analysis. In the multivariate analysis of BP comparison between groups, age, sex, body mass index, estimated glomerular filtration rate (eGFR), presence of diabetes mellitus, presence of cardiovascular or cerebrovascular disease, duration of exercise per week, duration of antihypertensive medications, amount of alcohol consumption per day, diet control, smoking status, and number of antihypertensive drug classes were adjusted as covariates with Bonferroni correction. The target BP achievement rate among the three groups was compared using the chi-square test. In the comparison of target BP achievement rates between HBPM(+)-R(+) vs. HBPM(−) groups and HBPM(+)-R(+) vs. HBPM(+)-R(−) groups, *p*-values < 0.0167 were considered statistically significant according to the Bonferroni correction.

For the effect of reporting the measured home BP to physicians on target BP achievement rate among the patients measuring home BP, multivariate logistic regression analysis was carried out and adjusted for age, sex, body mass index, eGFR, presence of diabetes mellitus, presence of cardiovascular or cerebrovascular disease, duration of exercise per week, duration of antihypertensive medications, amount of alcohol consumption per day, diet control, smoking status, and number of antihypertensive drug classes.

## Results

Among the 701 patients recruited, 673 patients were included, and 28 were excluded for the following reasons: 26 patients withdrew informed consent; one patient met the exclusion criteria (arrhythmia); and one patient did not meet the inclusion criteria (discontinuation of antihypertensive drug). Among the patients who responded to measuring BP at home (*n* = 278), no difference in the duration of home BP measurements between patients who reported and those who did not report the measured BP to physicians existed. However, the frequency of home BP measurements between patients who reported vs. those who did not report measured BP to physicians was significantly different (*p* < 0.001). Patients who reported measured home BP to physicians showed a higher frequency of home BP measurement than those who did not report measured BP to physicians (Table 1).

**TABLE 1:** Duration and frequency of home blood pressure measurements in patients who responded to measure home blood pressure.

	All	R(−)	R(+)	*p*
*n*	278	135	143	
**Duration of home BP measurement**				
<6 months	15 (5.4)	7 (5.1)	8 (5.6)	1.000
≥6 months	263 (94.6)	129 (94.9)	134 (94.4)	
**Frequency of home BP measurements**				
Everyday	46 (16.5)	13 (9.6)	33 (23.2)	<0.001
3–5 days/week	41 (14.7)	12 (8.8)	29 (20.4)	
1–2 days/week	57 (20.5)	21 (15.4)	36 (25.4)	
1–3 days/month	76 (27.3)	41 (30.1)	35 (24.6)	
<1 days/month	58 (20.9)	49 (36.0)	9 (6.3)	

R(−), not report blood pressure measured at home to physicians; R(+), report blood pressure measured at home to physicians. Duration of home BP measurement: period of home BP measurements from the survey day.

Based on the definition of proper home BP measurements described in the “Materials and methods” section, 467 were assigned to the HBPM(−) group, 81 to the HBPM(+)-R(−) group, and 125 to the HBPM(+)-R(+) group (Table 2). The HBPM(−) group had a higher BMI than the HBPM(+) group (*P* = 0.016). There was no difference between the groups in mean age, sex, body mass index, eGFR, smoking status, alcohol consumption, duration of exercise, and effort to control diet. The prevalence of diabetes mellitus and cardiovascular disease, number of antihypertensive drugs, duration of antihypertensive medications, classes of antihypertensive drugs, and self-reported compliance to antihypertensive medications also did not differ between the groups. There was no difference in eGFR, prevalence of chronic kidney disease (eGFR < 60 ml/min/1.73 m^2^), urine albumin-to-creatinine ratio, and prevalence of left ventricular hypertrophy by voltage of Sokolov-Lyon criteria between the groups (Table 2).

**TABLE 2:** Demographic and clinical characteristics of the study population.

	All	HBPM(−)	HBPM(+)				
			All	HBPM(+)-R(−)	HBPM(+)-R(+)	*p* ^a^	*p* ^b^
*n*	673	467	206	81	125		
Age, years	64.9 ± 9.3	65.4 ± 9.3	63.9 ± 9,1	65.1 ± 9.9	63.1 ± 8.5	0.059	0.053
**Sex**							
Male, %	51.3	50.1	53.9	53.1	54.4	0.366	0.654
Female, %	48.7	49.9	46.1	46.9	45.6		
BMI, kg/m^2^	25.3 ± 3.0	25.5 ± 3.0	24.9 ± 2.9	25.0 ± 3.3	24.9 ± 2.6	0.016	0.052
Smoking, %	8.0	8.8	6.3	7.4	5.6	0.277	0.497
Amount of alcohol drinking, gr/day	5.7 ± 12.8	5.7 ± 12.5	5.7 ± 13.6	6.4 ± 18.6	5.3 ± 9.1	0.988	0.829
Duration of exercise, h/week	4.2 ± 4.7	4.2 ± 4.9	4.3 ± 4.3	4.8 ± 4.6	4.0 ± 4.0	0.765	0.454
Diet control for hypertension, %	16.3	15.4	18.4	19.8	17.6	0.327	0.570
Diabetes mellitus, %	24.1	25.3	21.4	23.5	20.0	0.274	0.469
Cardiovascular disease, %	16.8	15.8	18.9	24.7	15.2	0.324	0.126
Number of antihypertensive drugs, *n*	1.7 ± 0.7	1.7 ± 0.7	1.7 ± 0.7	1.7 ± 0.7	1.8 ± 0.7	0.786	0.828
**Antihypertensive drugs**							
ACE inhibitors, %	1.3	1.3	1.5	2.5	0.8	0.858	0.586
ARBs, %	74.3	74.7	73.3	65.4	78.4	0.695	0.106
Beta blockers, %	13.4	13.3	13.6	12.3	14.4	0.912	0.909
Calcium channel blockers, %	63.0	62.7	63.6	70.4	59.2	0.833	0.262
Diuretics, %	20.5	20.1	21.4	18.5	23.2	0.716	0.672
Doses of antihypertensive drugs, standard dose	1.93 ± 1.04	1.93 ± 1,04	1.92 ± 1.02	1.94 ± 1.12	1.91 ± 0.95	0.909	0.972
Duration of antihypertensive medications, years	10.4 ± 7.4	10.4 ± 7.1	10.6 ± 8.0	11.4 ± 8.3	10.1 ± 7.8	0.710	0.434
Compliance to medication, %	98.4 ± 6.3	98.1 ± 7.2	99.1 ± 3.1	99.2 ± 2.9	99.0 ± 3.2	0.013	0.168
eGFR, ml/min/1.73 m^2^	86.4 ± 14.3	85.9 ± 14.3	87.6 ± 14.1	86.0 ± 16.2	88.6 ± 12.6	0.162	0.166
CKD, *n* (%)	30 (4.5)	25 (5.4)	5 (2.4)	4 (4.9)	1 (0.8)	0.090	0.088
UACR	33.9 ± 149.9	32.9 ± 130.1	36.1 ± 187.4	56.3 ± 288.8	23.1 ± 61.8	0.290	0.799
LVH, *n* (%)	59 (9.3)	43 (9.7)	16 (8.3)	9 (11.7)	7 (6.0)	0.577	0.355

*p*^a^, comparison between HBPM(−) and HBPM(+) groups by *t*-test or Chi-square test. *p*^b^, comparison between HBPM(−), HBPM(+)-R(−), and HBPM(+)-R(+) groups by ANOVA or Chi-square test. Data are expressed as mean ± standard deviation, percent, or number and percent in parentheses. HBPM(−), not measure blood pressure at home based on our definition; HBPM(+), measure home BP properly based on our definition; HBPM(+)-R(−), measure blood pressure at home properly but not report measured home blood pressure to physicians; HBPM(+)-R(+), measure blood pressure at home properly and report measured home blood pressure to physicians; ACE, angiotensin-converting enzyme; ARB, angiotensin receptor blocker; eGFR, estimated glomerular filtration rate; CKD, chronic kidney disease (eGFR < 60 ml/min/1.73 m^2^); UACR, urine albumin-to-creatinine ratio; LVH, left ventricular hypertrophy by voltage of Sokolov-Lyon criteria.

**FIGURE 1:**
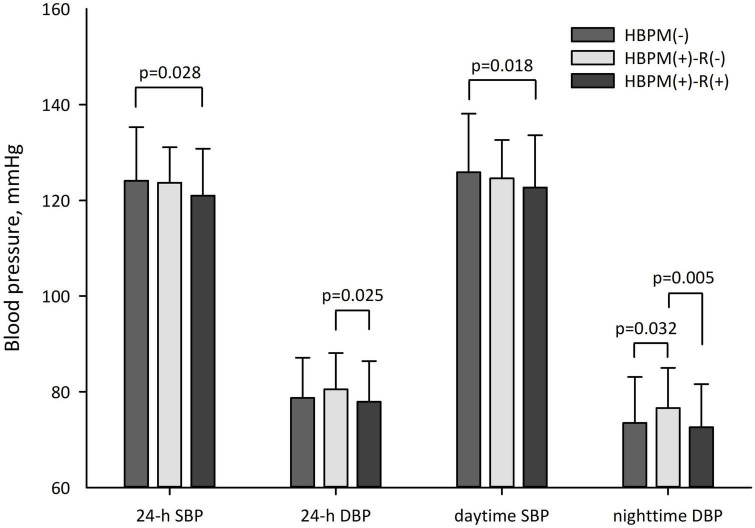
Ambulatory blood pressure difference between groups. *P*-values were obtained by multivariate analysis.

Table 3 shows a comparison of office and ambulatory BP between groups. Office SBP and DBP were not different between the HBPM(−) and HBPM(+) groups. The HBPM(+) group had significantly lower 24-h and daytime SBP than the HBPM(−) group (*p* = 0.014 and *p* = 0.007, respectively), and these differences were persistent in multivariate analysis (*p* = 0.023 and *p* = 0.006, respectively, Figure 1).

**TABLE 3:** Blood pressure differences.

	HBPM(−)	HBPM(+)			HBPM(−) vs. HBPM(+)		HBPM(−) vs. HBPM(+)-R(−) vs. HBPM(+)-R(+)	
		All	HBPM(+)-R(−)	HBPM(+)-R(+)	*p*	*P*_*multivariate*_	*P*_*univariate*_	*P*_*multivariate*_
*n*	467	206	81	125				
Office SBP	128.5 ± 11.4	126.9 ± 9.7	128.6 ± 9.3	125.8 ± 9.9^a^	0.071	0.134	0.041	0.153
Office DBP	77.9 ± 7.8	78.0 ± 7.7	79.0 ± 7.5	77.3 ± 7.8	0.869	0.565	0.288	0.085
*n*	449	201	81	120				
24-h SBP	124.1 ± 11.2	122.1 ± 9.0	123.7 ± 7.4	121.0 ± 9.8^b*^	0.014	0.023	0.017	0.033
24-h DBP	78.7 ± 8.4	78.9 ± 8.2	80.5 ± 7.6	77.9 ± 8.5^†^	0.678	0.695	0.085	0.029
Daytime SBP	125.9 ± 12.2	123.4 ± 9.8	124.6 ± 8.0	122.7 ± 10.9^c‡^	0.007	0.006	0.024	0.016
Daytime DBP	80.1 ± 9.3	80.2 ± 9.0	81.5 ± 8.2	79.3 ± 9.5	0.978	0.333	0.262	0.059
Nighttime SBP	117.6 ± 13.6	116.8 ± 11.7	119.9 ± 10.8	114.8 ± 11.8^d^	0.437	0.557	0.017	0.060
Nighttime DBP	73.5 ± 9.6	74.2 ± 9.0	76.6 ± 8.4^e§^	72.6 ± 9.0	0.356	0.649	0.007	0.006

Data are expressed as mean ± standard deviation. HBPM(−), not measure blood pressure at home based on our definition; HBPM(+), measure home BP properly based on our definition; HBPM(+)-R(−), measure blood pressure at home properly but not report measured home blood pressure to physicians; HBPM(+)-R(+), measure blood pressure at home properly and report measured home blood pressure to physicians; SBP, systolic blood pressure; DBP, diastolic blood pressure. *P_univariate_*, Univariate analysis of variance (ANOVA) with Tukey’s or Games Howell *post-hoc* analysis:

^a^*p* = 0.035 HBPM(+)-R(+) vs. HBPM(−).

^b^*p* = 0.009 HBPM(+)-R(+) vs. HBPM(−).

^c^*p* = 0.016 HBPM(+)-R(+) vs. HBPM(−).

^d^*p* = 0.005 HBPM(+)-R(+) vs. HBPM(+)-R(−).

^e^*p* = 0.015 HBPM(+)-R(−) vs. HBPM(−) and *p* = 0.008 HBPM(+)-R(+) vs. HBPM(+)-R(+). *P_multivariate_*, Multivariate analysis (ANCOVA with Bonferroni correction) adjusted for age, gender, body mass index, estimated glomerular filtration rate (eGFR), presence of diabetes mellitus, presence of cardiovascular or cerebrovascular disease, duration of exercise per week, duration of antihypertensive medications, amount alcohol drinking per day, diet control or not, smoking status, number of antihypertensive drug classes as a covariate with a Bonferroni correction.

*p = 0.028 HBPM(+)-R(+) vs. HBPM(−).

^†^*p* = 0.025 HBPM(+)-R(+) vs. HBPM(+)-R(−).

^‡^*p* = 0.018 HBPM(+)-R(+) vs. HBPM(−).

^§^*p* = 0.005 HBPM(+)-R(−) vs. HBPM(+)-R(+) and *p* = 0.032 HBPM(+)-R(−) vs. HBPM(−).

The HBPM(+)-R(+) group had lower office SBP (*p* = 0.035), 24-h SBP (*p* = 0.009), and daytime SBP (*p* = 0.016) than the HBPM(−) group and lower nighttime SBP (*p* = 0.005) and diastolic DBP (*p* = 0.008) than the HBPM(+)-R(−) group. The HBPM(+)-R(−) group had a higher nighttime DBP than the HBPM(−) group (*p* = 0.015). In the multivariate analysis, the difference in 24-h SBP and daytime SBP between the HBPM(+)-R(+) and HBPM(−) groups remained significant (*p* = 0.028 and *p* = 0.018, respectively, Figure 1). The significant difference in nighttime DBP between the HBPM(+)-R(−) and HBPM(+)-R(+) groups and between the HBPM(+)-R(−) and HBPM (−) groups was persistent (*p* = 0.005 and *p* = 0.032, respectively, Figure 1). The 24-h DBP of the HBPM(+)-R(+) group was lower than that of the HBPM(+)-R(−) group (*p* = 0.025, Figure 1).

**FIGURE 2:**
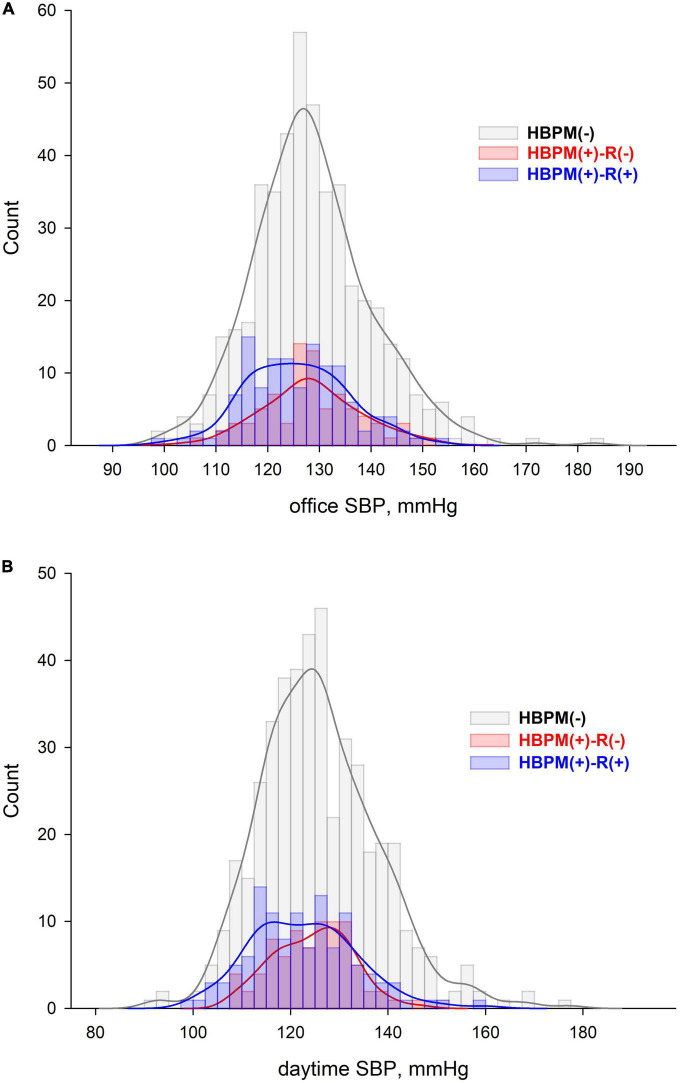
Histogram and Kernel density of **(A)** office and **(B)** daytime systolic blood pressure distribution in each group.

Histograms revealed broad peak of office SBP with a more frequent office SBP around 120 mmHg in the HBPM(+)-R(+) group compared with that of other groups (Figure 2A). Daytime SBP of HBPM(+)-R(+) group showed bimodal distribution with a peak below 120 mmHg (Figure 2B).

Table 4 shows comparisons of target BP achievement rates between groups. The HBPM(+) group had a better target BP achievement rate only in daytime SBP compared with the HBPM(−) group (74.6% vs. 66.4%, *p* = 0.036, respectively). When the HBPM (+) group was classified according to reporting measured home BP to the physician, the target achievement rates of office BP were 44.8% for the HBPM(−) group, 44.4% for the HBPM(+)-R(−) group, and 56.8% for the HBPM(+)-R(+) group. Although the target achievement rates of office BP of the HBPM(+)-R(+) group was the highest, the statistical difference between groups was marginally not different (*p* = 0.050). Analysis between the two groups showed higher target achievement rates of office BP in the HBPM(+)-R(+) group than in the HBPM(−) group (*p* = 0.0166). The target achievement rates of 24-h, daytime, and nighttime BP were not statistically different between the groups.

**TABLE 4:** Control rates of blood pressure.

	HBPM(−)	HBPM(+)			HBPM(−) vs. HBPM(+)	HBPM(−) vs. HBPM(+)-R(−) vs. HBPM(+)-R(+)		
		All	HBPM(+)-R−)	HBPM(+)-R(+)	*p*	*p* ^a^	*p* ^b^	*p* ^c^
Office BP, *n* (%)	209 (44.8)	107 (51.9)	36 (44.4)	71 (56.8)	0.085	0.050	0.083	0.017
24-h BP, %	125 (27.8)	54 (26.9)	17 (21.0)	37 (30.8)	0.797	0.299	0.145	0.569
Daytime BP, %	186 (41.4)	88 (43.8)	34 (42.0)	54 (45.0)	0.574	0.780	0.772	0.533
Nighttime BP, %	65 (14.5)	24 (11.9)	5 (6.2)	19 (15.8)	0.385	0.102	0.046	0.772
Office SBP, %	282 (60.4)	134 (65.0)	51 (63.0)	83 (66.4)	0.251	0.458	0.613	0.219
Office DBP, %	282 (60.4)	133 (64.6)	45 (55.6)	88 (70.4)	0.304	0.060	0.030	0.040
24-h SBP, %	256 (57.0)	125 (62.2)	43 (53.1)	82 (68.3)	0.216	0.046	0.029	0.025
24-h DBP, %	157 (35.0)	66 (32.8)	22 (27.2)	44 (36.7)	0.597	0.330	0.159	0.729
Daytime SBP, %	298 (66.4)	150 (74.6)	60 (74.1)	90 (75.0)	0.036	0.109	0.882	0.071
Daytime DBP, %	219 (48.8)	100 (49.8)	36 (44.4)	64 (53.3)	0.818	0.453	0.216	0.375
Nighttime SBP, %	140 (31.2)	59 (29.4)	15 (18.5)	44 (36.7)	0.640	0.021	0.006	0.254
Nighttime DBP, %	89 (19.8)	31 (15.4)	6 (7.4)	25 (20.8)	0.182	0.023	0.010	0.806

Data are expressed as number and percent in parentheses. *p*^a^, Chi-square test between three groups. *p*^b^, Chi-square test between HBPM(+)-R(+) and HBPM(+)-R(−). *p*^c^, Chi-square test between HBPM(+)-R(+) and HBPM(−) groups. HBPM(−), not measure blood pressure at home based on our definition; HBPM(+), measure home BP properly based on our definition; HBPM(+)-R(−), measure blood pressure at home properly but not report measured home blood pressure to physicians; HBPM(+)-R(+), measure blood pressure at home properly and report measured home blood pressure to physicians; SBP, systolic blood pressure; DBP, diastolic blood pressure.

In the analysis of target achievement rates of SBP and DBP, there was a significant difference between the groups in the target achievement rate of 24-h SBP (*p* = 0.046), nighttime SBP (*p* = 0.021), and nighttime DBP (*p* = 0.023). The target achievement rates of office DBP and 24-h SBP of the HBPM(+)-R(+) group were higher than those of the HBPM(+)-R(−) group (70.4 vs. 55.6%, *p* = 0.030 and 68.3 vs. 53.1%, *p* = 0.029, respectively). However, the difference was not statistically significant with the Bonferroni correction. The target achievement rates of nighttime SBP and DBP in the HBPM(+)-R(+) group were significantly higher than those in the HBPM(+)-R(−) group (36.7 vs. 18.5%, *p* = 0.006 and 20.8 vs. 7.4%, *p* = 0.010, respectively). The target achievement rates of office DBP and 24-h SBP between the HBPM(+)-R(+) and HBPM(−) groups were not statistically significant with the Bonferroni correction (*p* = 0.048 and *p* = 0.028, respectively). The target BP achievement rate according to the ESC/ESH hypertension guidelines is presented in the Supplementary Table 1.

Among the patients measuring home BP, the adjusted odds ratios for the target achievement rate of 24-h and nighttime BP in the HBPM(+)-R(+) group were 2.233 (95% CI, 1.037–4.807) and 3.658 (95% CI, 1.115–11.994), respectively, compared with those of the HBPM(+)-R(−) group.

## Discussion

In our study, patients measuring home BP had lower 24-h and daytime SBPs and higher target achievement rates of daytime SBP than those who did not measure home BP. Not all patients measuring home BP reported the measured home BP to their physicians. Among the patients measuring home BP, 60.7% reported measured home BP, whereas 39.3% did not. Reporting measured home BP to the treating physicians showed a marked effect on BP control. Patients who measured home BP and reported to their physicians had lower 24-h and daytime SBPs and a higher target achievement rates of office BP than those who did not measure home BP. Patients who measured home BP and reported to their physicians had lower 24-h and nighttime DBPs and better target achievement rates of nighttime SBP and DBP than those who measured home BP but did not report it to physicians. The results of our study suggest that reporting measured home BP to physicians rather than just measuring it seems to control BP more effectively.

Prospective randomized controlled studies (8–10) and meta-analyses (11–13) have shown the efficacy of home BP measurement in lowering BP. Contrastingly, studies for the effectiveness of home BP measurement on the treatment of hypertension in the real world are sparse and show controversial results (15, 16). In a study in France (15), although the researches did not investigate whether home BP was measured, patients owning home BP measurement devices had similar SBP but lower DBP compared with those not owning home BP measurement devices among those who were aware of their hypertension. In the National Health and Nutrition Examination Survey (NHANES) in the United States (16), patients who received a physician recommendation and measured home BP had no difference in SBP but showed higher DBP compared with those who neither received recommendations nor measured home BP.

The reason for the controversial results in the real world could be attributed to the efficacy-effectiveness gap (17). Most previous randomized controlled studies on the efficacy of home BP measurements were conducted under the condition that the measured home BP was reported to the physicians and reflected in the treatment of hypertension. However, many patients in the real world do not share their measured home BP with physicians, as shown in our and other studies (19, 22). In our study, 39.3% of patients among those measuring home BP did not report their measured home BP to physicians; therefore, BP measured at home was not reflected in the treatment of hypertension. In a survey study in Japan, 23.8% of patients among those measuring and recording home BP did not report the recorded home BP to their physicians (19). A survey study in Canada found that 68% of the surveyed patients did not or barely took the records of home BP to their physicians (22). Even if a patient measures their home BP, it may be meaningless if it is not reflected in the treatment of hypertension. In contrast to previous studies, we focused on whether reporting measured home BP to physicians affects BP control, which has not been previously evaluated. The results of our research indicated that reporting measured home BP to physicians is an important factor contributing to the efficacy-effectiveness gap in real-world patients, emphasizing the importance of interaction between patients and physicians.

In addition to patient factors contributing to the insufficient effectiveness of home BP measurement in the treatment of hypertension in the real world, physician factors should be considered. Many physicians questioned the accuracy and value of home-measured BP (20, 25). In a survey in The United States, only 29.7% of patients with hypertension received a recommendation for home BP measurement (19). A low rate of physician recommendation to measure home BP may contribute to the low effectiveness of home BP measurements in the real world. Our study did not investigate whether physicians recommended home BP measurements. Instead, only 59 patients (8.8%) in our study received recommendations to purchase a home BP measurement device from their physicians (Question 3 of Supplementary material, data not shown). Considering the similar rate of home BP measurement in our study (41.3%) to that in The United States survey study (43.7%) (16) and 48.6% of non-reporting home BP to physicians, physician recommendations to measure home BP are also expected to be low. Importantly, the more frequent distribution of lower BP in the HBPM(+)-R(+) group suggests a potential role of home BP measurement and reporting to physicians in overcoming the therapeutic inertia in the real world (11).

There is a long road to implementing home BP measurement in the diagnosis and treatment of hypertension (21). To improve the implementation of home BP measurement in real-world clinical practice, a systematic approach is needed from the perspective of patients, physicians, and healthcare providers. Many guidelines recommend using home BP measurements in diagnosing and treating hypertension (6, 7, 14). Despite the efforts of many societies, home BP measurements do not seem optimal or effective in the real world. Since the participant recruiting advertisement for our study included the title of the study: “Study for the effect of home BP measurement in the treatment of hypertension”, it is assumed that more people interested in BP control using home BP measurements may have participated in the study. Accordingly, fewer patients are expected to engage in home BP measurement in the real world. The fact that many patients in our study did not report their BP measured at home to their physicians also means that many of their physicians were not interested in home BP measurement and did not ask the patients to show their BP measured at home. In a survey conducted among Korean physicians, only 29% prioritized home BP measurement for new hypertension diagnosis and only 6.6% fully explained home BP measurement protocols to their patients during visits (25). This indicated that providing education and materials for the method of using home BP measurement to general physicians is very limited in clinical practice.

The strength of our study is that the BP level and control status were assessed using ambulatory BP. Office BP measurements are prone to white-coat and masked effects. Home BP has the advantage of being able to diagnose white-coat and masked uncontrolled hypertension. Home-measured BP was closer to ambulatory BP than office BP. Therefore, the results of our study may better reflect the effects of home BP measurements.

Our study also had some limitations. First, accurate measurement of home BP is critical for the proper management of hypertension and may prevent progression of cardiovascular disease (26). As per proper home BP measurements, we did not consider whether the home BP was measured according to the guidelines (i.e., in a quiet room with a comfortable temperature; proper posture during measurement; no caffeine, smoking, or exercise 30 min before measurement; two or more measurements in the morning and evening; 1–2 min interval between consecutive measurements; and use of validated devices) in the study (6, 7, 14). In contrast to a controlled study, many patients in the real world do not receive materials or education for the proper measurement of home BP. In our study, 33.5% of patients measuring home BP followed the guideline recommendations (data not shown). If we regarded patients who measured home BP following guideline recommendations as those who measured home BP properly, the number of patients was too small to analyze the effectiveness of home BP measurement on BP control in our study population. Second, whether the patients reported measured home BP properly and whether reported home BP was used in the treatment of hypertension by the treating physicians were of concern. In our study, patients reported home-measure BP to physicians either verbally, *via* showing records on a measurement record sheet, or *via* showing values stored in the memory of home BP measurement device. In addition, reporting to a physician does not necessarily mean that the home-measured BP was used in treating hypertension by physicians. This study was unable to investigate whether physicians treating hypertension used the reported home BP in the treatment. All the issues concerning the accurate measurement of home BP, reporting method, using home-measure BP by the treating physician were very complicated to inclusively consider in this study. Therefore, in this study, we only evaluated whether reporting home-measured BP to the treating physicians had a different effect on BP control. Third, we measured basic markers of hypertension mediated organ damage (7), and there was no difference between all groups. Although we did not evaluate the presence of hypertensive retinopathy, carotid plaque or stenosis, aortic disease and peripheral arterial disease, the possibility of difference between the groups was considered low because the prevalence of known cardiovascular disease showed no statistical difference. Finally, we were not able to explain the mechanism for the lower level and higher control rate of ambulatory BP in patients who measured and reported home BP, regarding no difference in lifestyle factors, the number and classes of antihypertensive medications, and self-reported compliance between groups. Therefore, future studies should further investigate the aforementioned aspects.

Despite these limitations, our study reported an important real-world situation in which many patients measure home BP but fail to report it to their treating physicians, a behavior interfering the effective treatment of hypertension.

## Conclusion

We demonstrated that patients with hypertension should not only measure home BP but also report these to the treating physician for the home BP measurements to be effective in managing hypertension in the real world. Encouraging home BP measurement and sharing the measured home BP with a physician will improve the treatment effect of hypertension in the real world. Further studies on the status of home BP measurement and its effects will help further establish a policy that incorporates home BP measurements into routine clinical practice.

## Data availability statement

The raw data supporting the conclusions of this article will be made available by the authors, without undue reservation.

## Ethics statement

The studies involving human participants were reviewed and approved by Institutional Review Board of Dongguk University Ilsan Hospital. The patients/participants provided their written informed consent to participate in this study.

## Author contributions

MYR performed material preparation and data collection and wrote the first draft of the manuscript. HYK and MYR performed the statistical analysis. All authors reviewed and commented on previous versions of the manuscript, and contributed to the study conception, read, and approved the final manuscript.
