# Supplementary material for: Home blood pressure measurement for hypertension management in the real world: Do not just measure, but share with your physician
Source: Front Cardiovasc Med. 2023 Jan 18;10:1103216. doi: 10.3389/fcvm.2023.1103216 (PMC9889357; doi:10.3389/fcvm.2023.1103216)
Supplement: Supplementary file 1 [file Data_Sheet_1.PDF]

Questionnaires for the status of home blood pressure measurements

(1) Do you have a home BP measurement device? “yes” or “no”. If you have, the manufacturer, model name, and date of production?

(2) Do you measure home BP? “yes” or “no”

(3) Did your physician recommend buying a home BP device? “yes” or “no”.

(4) When did you start to measure home BP from the survey?

  (a) within one month

  (b) between 1 and 3 months

  (c) between 3 and 6 months

  (d) between 6 months and one year

  (e) more than one year

(5) The frequency of home BP measurements

  (a) every day

  (b) 3 to 5 days per week

  (c) 1 to 2 days per week

  (d) 1 to 3 days per month

  (e) once every few months

(6) When do you measure home BP?

  (a) in the morning

  (b) the day

  (c) in the evening

  (d) any time

(7) how many consecutive readings do you take during home BP measurements?

  (a) once

  (b) twice

  (c) triple

  (d) more than 4

(8) how long do you take a rest before home BP measurements?

    (a) immediate
    (b) 1 minute
    (c) 2 to 5 minutes
    (d) more than 5 minutes

(9) the interval between measurements

    (a) immediate
    (b) 1 minute
    (c) 2 to 5 minutes
    (d) more than 5 minutes

(10) do you measure home BP within 30 minutes after coffee drinking? “yes” or “no”

(11) do you measure home BP within 30 minutes after doing exercise? “yes” or “no”

(12) do you measure home BP within 30 minutes after smoking? “yes” or “no”

(13) do you use an appropriate size cuff for your arm? “yes” or “no”

(14) do you record the measured home BP? “yes” or “no”

(15) do you report the measured home BP to your physician? “yes” or “no”

Since Korean is translated into English, there may be slight differences.
