# Supplementary material for: Home blood pressure measurement for hypertension management in the real world: Do not just measure, but share with your physician
Source: Front Cardiovasc Med. 2023 Jan 18;10:1103216. doi: 10.3389/fcvm.2023.1103216 (PMC9889357; doi:10.3389/fcvm.2023.1103216)
Supplement: Supplementary file 2 [file Table_1.docx]

Supplementary Table 1. ESC/ESH target blood pressure achievement rate

|  | HBPM(-) | HBPM(+) | | | HBPM(-) vs HBPM(+) | HBPM(-) vs HBPM(+)-R(-) vs HBPM(+)-R(+) | | |
| --- | --- | --- | --- | --- | --- | --- | --- | --- |
|  |  | All | HBPM(+)-R(-) | HBPM(+)-R(+) | p | p^a^ | p^b^ | p^c^ |
| Office BP, n (%) | 248 (53.1) | 120 (58.3) | 43 (53.1) | 77 (61.6) | 0.216 | 0.227 | 0.226 | 0.090 |
| 24-hr BP, % | 137 (30.5) | 57 (28.4) | 20 (24.7) | 37 (30.8) | 0.579 | 0.555 | 0.343 | 0.949 |
| Daytime BP, % | 196 (43.7) | 93 (46.3) | 36 (44.4) | 57 (47.5) | 0.535 | 0.753 | 0.670 | 0.451 |
| Nighttime BP, % | 77 (17.1) | 29 (14.4) | 6 (7.4) | 23 (19.2) | 0.385 | 0.059 | 0.020 | 0.606 |
|  |  |  |  |  |  |  |  |  |
| Office SBP, % | 345 (73.9) | 157 (76.2) | 66 (81.5) | 91 (72.8) | 0.521 | 0.306 | 0.153 | 0.808 |
| Office DBP, % | 282 (60.4) | 133 (64.3) | 45 (55.6) | 88 (70.4) | 0.304 | 0.606 | 0.030 | 0.040 |
| 24-hr SBP, % | 296 (65.9) | 138 (68.7) | 53 (65.4) | 85 (70.8) | 0.494 | 0.576 | 0.418 | 0.310 |
| 24-hr DBP, % | 157 (35.0) | 66 (32.8) | 22 (27.2) | 44 (36.7) | 0.597 | 0.330 | 0.159 | 0.729 |
| Daytime SBP, % | 321 (71.5) | 159 (79.1) | 66 (81.5) | 93 (77.5) | 0.041 | 0.102 | 0.496 | 0.189 |
| Daytime DBP, % | 219 (48.8) | 100 (49.8) | 36 (44.4) | 64 (53.3) | 0.818 | 0.453 | 0.216 | 0.375 |
| Nighttime SBP, % | 199 (44.3) | 86 (42.8) | 27 (33.3) | 59 (49.2) | 0.716 | 0.080 | 0.026 | 0.344 |
| Nighttime DBP, % | 89 (19.8) | 31 (15.4) | 6 (7.4) | 25 (20.8) | 0.182 | 0.023 | 0.010 | 0.806 |

Data are expressed as number and percent in parentheses.

p^a^, Chi-square test between three groups; p^b^, Chi-square test between HBPM(+)-R(+) and HBPM(+)-R(-); p^c^, Chi-square test between HBPM(+)-R(+) and HBPM(-) groups

HBPM(-), not measure blood pressure at home based on our definition; HBPM(+), measure home BP properly based on our definition; HBPM(+)-R(-), measure blood pressure at home properly but not report measured home blood pressure to physicians; HBPM(+)-R(+), measure blood pressure at home properly and report measured home blood pressure to physicians; SBP, systolic blood pressure; DBP, diastolic blood pressure;
